# Shear Performance of Horizontal Joints in Short Precast Concrete Columns with Sleeve Grouted Connections under Cyclic Loading

**DOI:** 10.1371/journal.pone.0165988

**Published:** 2016-11-18

**Authors:** Bo Feng, Feng Xiong, Bingyu Liu, Jiang Chen, Yiping Zhang

**Affiliations:** 1 College of Architecture & Environment, Sichuan University, Chengdu, Sichuan Province, China; 2 Sichuan Provincial Architectural Design and Research Institute, Chengdu, Sichuan Province, China; Beihang University, CHINA

## Abstract

In this study, two short precast concrete columns and two cast-in-situ concrete columns were tested under cyclic loads. It was shown that the sleeve grouted connection was equivalent to the cast-in-situ connections for short columns when the axial compression ratio was 0.6. In order to determine the influence of the axial compression ratio and the shear-span ratio on the shear capacity of the horizontal joint, a FE model was established and verified. The analysis showed that the axial compression ratio is advantageous to the joint and the shear capacity of the horizontal joint increases with increase of the shear-span ratio. Based on the results, the methods used to estimate the shear capacity of horizontal joints in the Chinese Specification and the Japanese Guidelines are discussed and it was found that both overestimated the shear capacity of the horizontal joint. In addition, the Chinese Specification failed to consider the influence of the shear-span ratio.

## Introduction

With the development of industrialization, precast concrete structures are currently used due to the advantages of lower cost, better quality, more rapid construction, and lower impact on environment etc. in comparison with cast-in-situ structures [1, 2]. The connection between the elements of a precast concrete structure is always a research emphasis, as it determines the integrity of the whole structure and influences the performance, manufacture, erection and maintenance [3].The sleeve grouted connection has been proven to be an effective monolithic joint and can ensure that a precast structure works as a cast-in-situ structure[4,5], so it is widely used worldwide and is recommended in the new Chinese Prefabricated Specifications[6].

Two types of shear failure modes can occur in short precast concrete columns. Shear failure with diagonal cracks passing through the column, defined as a diagonal shear failure in this paper, is the same type of failure mode as in the cast-in-situ column. A second type of shear failure might occur along the horizontal joint between the upper and downside precast columns, defined as a horizontal shear failure here. The horizontal joint is naturally a weak plane for precast columns, no matter what the connection is. Therefore, horizontal shear failure can occur prior to diagonal shear failure, especially for short columns with shear-span ratios less than 2.0 that are frequently used in high-rise precast concrete frame structures due to the large column section size.

According to ref [7], the horizontal shear capacity is composed of friction along the joint interface, dowel action of the rebar, and the cohesive force provided by joint-filled materials. The cohesive force is relatively lower compared with the other effects; therefore, it is usually ignored in the design process. Since the cross-section area of the longitudinal reinforced bar is usually determined by the bending capacity, the approach usually adapted to improve the horizontal shear capacity is to increase the friction, such as having a rough connection interface, or setting shear keys at the connection interface. Rizkalla et.al [8] found that the presence of shear keys enhanced the shear capacity.

Some scholars[9–11] have proposed empirical and semi-empirical equations to calculate the horizontal shear strength. However, most of the equations were established based on the results of push-off tests, which didn’t take into account the influence of the shear-span ratio and the cyclic loads. So the shear strengths estimated by such equations may have a certain error compared to the actual shear strengths of the horizontal joints. On the other hand, the equations to calculate the horizontal shear strength suggested by various codes are quite different, such as the friction between concrete surfaces and the dowel action of the rebar are considered simultaneously in the Chinese Specification[6] and the shear bearing capacity is equal to their summation; however, in the Japanese Guideline[12], the bearing capacity takes the larger value of the two load paths.

To investigate the shear performance of horizontal joints for short precast columns with sleeve grouted connections, and explore the conditions under which shear failure occurs along the joint, two short cast-in-situ concrete columns and two short precast concrete columns were tested under cyclic horizontal loading. However, no joint failures were observed during the tests. The test results indicated that there were no differences in the precast and cast-in-situ specimens in the failure mode. Therefore, a numerical analysis was established to study the horizontal shear failure further. Based on the results of numerical analysis, the horizontal shear failure was investigated, and the equations for the horizontal joint shear strength in the Chinese Specification[6]and the Japanese Guideline[12]were compared and evaluated.

## Test program and results

### Design of test specimens

**Fig 1:**
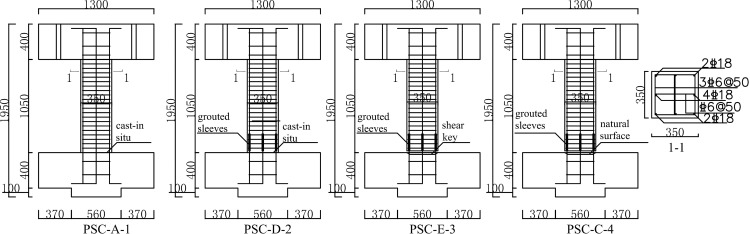
Details of the specimens (unit: mm).

Four short column specimens were designed: two cast-in-situ specimens and two precast specimens, designated as PSC-A-1, PSC-D-2, PSC-E-3, and PSC-C-4. Specimen PSC-A-1, as the baseline for this group of specimens, was a cast-in-situ specimen with continuous rebar. Specimen PSC-D-2 was also a cast-in-situ specimen but the rebar passing through the foundation to the column was connected with grouted sleeves targeting to investigate the influence of grouted sleeves on the shear performance. Specimen PSC-E-3 and PSC-C-4 were precast specimens with grouted-sleeve connected rebar. The horizontal joint interface of specimen PSC-C-4 was made as a natural surface, while specimen PSC-E-3 was dealt with as a shear key. The difference in specimens PSC-E-3 and PSC-C-4 was to observe the effect of different joint configurations. The shear-span ratio and axial compression ratio were fixed to1.5 and 0.6 (the limit value of axial compression ratio for the first-grade frame column is 0.65 as specified in reference[13]), respectively. The reinforcement ratio and stirrup ratio were 1.66% and 1.24% (the minimum stirrup ratio is 1.2% as specified in the reference[13]), respectively. Details of the specimens are shown in Fig 1. The strength grade of the concrete was C45 (cube crushing strength 45 MPa), and the grade of the rebar was HRB400 (yield strength 400 MPa).The compression strengths of the grouting material were 66 MPa for 3 days and 100 MPa for 30 days.

### Test procedure

**Fig 2:**
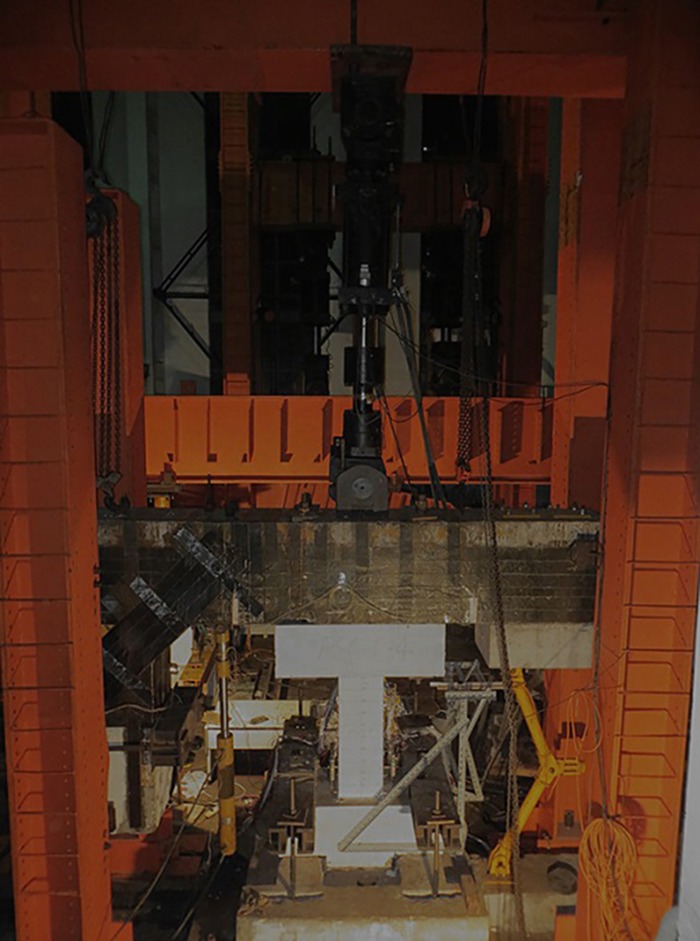
Test unit.

The test unit is shown in Fig 2. In order to simulate the constraint from the floor to the column, the lateral load was applied by an L-shaped beam, which had sufficient stiffness to constrain the in-plane rotation of the column. A constant vertical load scaled by 0.6*f*_c_*bh* was applied to simulate the vertical load from the upper structure, where *f*_c_ is the compressive strength of the concrete, and *b* and *h* are the breadth and depth of the column cross-section, respectively. The loading process of the lateral cyclic load was controlled by the displacement, actually the drift ratio, which is defined as the horizontal displacement divided by the column height. Drift ratios of 1/800, 1/400, 1/200, 1/100, 1/50, 1/25 were applied until the specimens lost bearing capacity, and each cycle was repeated thrice. When the bearing capacity degraded to 85% of the peak load [14] or the horizontal joint slid, it was considered that the specimen had reached the ultimate state.

### Specimen failure process

**Fig 3:**
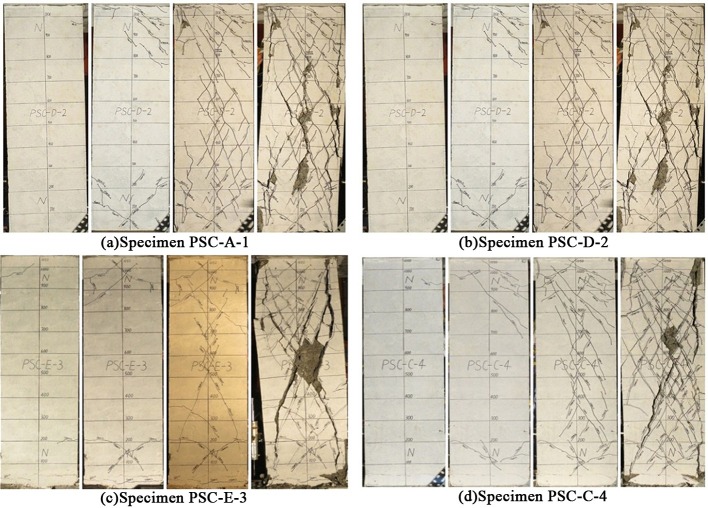
Crack developing process of the specimens.

Fig 3 shows the crack development of the specimens, which was found to be similar in all specimens. At the beginning of loading, bending (horizontal) cracks occurred at the top of the columns, and then developed diagonally in crossing cracks. With the increase of lateral displacement, the amount, length and width of the crossing cracks increased and a main crossing crack gradually formed at the centrality of the columns. At the end of loading, the concrete in the area of main crack spalled and the specimens showed clear diagonal shear failure. No obvious horizontal cracks or sliding along the horizontal joint were observed in specimen PSC-C-4. Besides, when the drift ratio was 1/800, a bending crack was observed in the horizontal joint of specimen PSC-E-3, but failed to develop continuously, and the specimen still failed ultimately by diagonal crossing cracks. Thus in this test, it can be concluded that the two precast specimens were damaged in a similar manner to the cast-in-situ specimens and failed in diagonal shear.

### Test results and discussions

**Fig 4:**
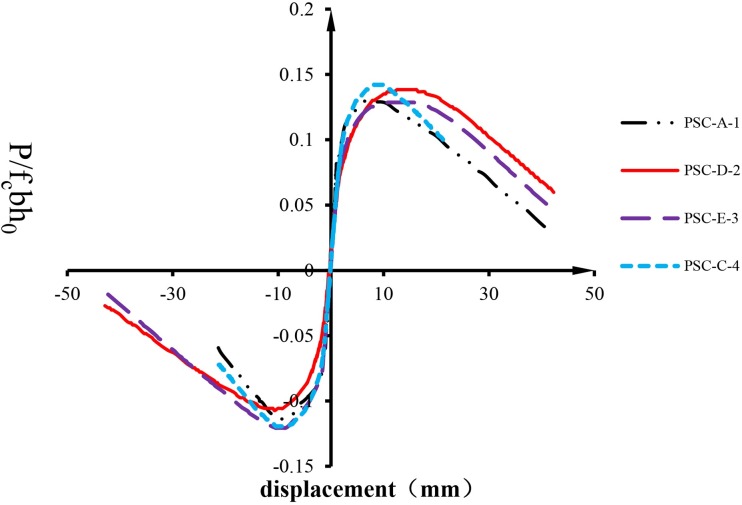
Dimensionless skeleton curves.

Skeleton curves, which are obtained by connecting the peak load of each loading cycle, reflect the capacity, stiffness and ductility of the structure, and are usually used to evaluate the performance of a structure under cyclic loading. Since the concrete strengths of the four specimens had slight differences, in order to eliminate the effects of the concrete strength, dimensionless skeleton curves of the four specimens are drawn in Fig 4, where the dimensionless lateral load is calculated as:

$$V_{\text{d}}=\frac{V}{f_{c}bh_{0}}$$ (1)

where *V*_d_ is the dimensionless lateral load; *V* is the applied lateral load; *f*_c_ is the concrete compression strength; *b* is the breadth of section; *h*_0_ is the effective depth of section.

It is observed in Fig 4 that all specimens experienced rising and descending stages, indicating a good deformation performance. When the specimens failed, specified as the lateral load dropping to 85% of the peak load, the drift ratios of PSC-D-2 and PSC-E-3 reached 1/50, while PSC-A-1 and PSC-C-4 reached 1/65. The peak loads of the skeleton curves were chosen to present the shear capacities of the short columns, which were the maximum values of the shear force the specimens undergoing during the overall loading process. Table 1 lists the shear capacities of all the specimens. It is observed that the dimensionless shear capacities of specimens PSC-A-1 and PSC-D-2 are nearly equal. It shows that the sleeve grouted connection which was used in the test is reliable and can ensure the force transfer between the longitudinal bars. Comparing specimen PSC-D-2 with specimens PSC-C-4 and PSC-E-3, it can be seen that the precast columns have not caused a significant decrease in the bearing capacity and the shear key in PSC-C-4 shows no indication of improving the shear capacity of the column significantly. Thus it is proven that the sleeve grouted connection, even on the natural interface without a shear key, can transfer the shear force under the test conditions.

**Table 1:** Shear Capacities of the Specimens.

Specimen	Shear capacity(kN)		Dimensionless shear capacity		Failure mode	Ultimate deformation(mm)	
	Positive direction	Negative direction	Positive direction	Negative direction		Positive direction	Negative direction
PSC-A-1	472.7	-406.9	0.112	0.097	Diagonal shear failure	17.1	14.1
PSC-D-2	504.2	-396.1	0.117	0.092	Diagonal shear failure	26.3	19.6
PSC-E-3	520.6	-493.3	0.112	0.106	Diagonal shear failure	25.4	17.5
PSC-C-4	517.7	-438.8	0.121	0.103	Diagonal shear failure	16.7	15

During the testing, the failure of the specimens occurred in a diagonal shear failure mode rather than horizontal failure mode along the joints. It means that the horizontal joints have a higher shear capacity than the column sections under the test conditions. In order to investigate the potential shear failure along the horizontal joint, nonlinear finite element models were established and parameter studies were carried out as described in the following sections.

## Nonlinear finite element analysis

### Material constitutive models

The finite element program ABAQUS was employed in this paper. The adopted stress-strain relationship for the concrete was that recommended in reference[15]. The uniaxial tension and compression stress-strain relationships are expressed as Eqs 2A–2E and 3A–3F, respectively.

$$\sigma =E_{\text{d}}\varepsilon$$ (2a)

$$E_{\text{d}}=E_{0}(1-d_{\text{t}})$$ (2b)

$$d_{\text{t}}=\{\begin{array}{ll} 1-\rho_{\text{t}}\left[1.2-0.2x^{5}\right] & x\leq 1\\ 1-\frac{\rho_{\text{t}}}{\alpha_{\text{t}}{\left(x-1\right)}^{1.7}+x} & x>1 \end{array}$$ (2c)

$$x=\frac{\varepsilon }{\varepsilon_{\text{t}}}$$ (2d)

$$\rho_{\text{t}}=\frac{f_{\text{t}}}{E_{0}\varepsilon_{\text{t}}}$$ (2e)

$$\sigma =E_{\text{d}}\varepsilon$$ (3a)

$$E_{\text{d}}=(1-d_{\text{c}})E_{0}$$ (3b)

$$d_{\text{c}}=\{\begin{array}{cc} 1-\frac{\rho_{\text{c}}n}{n-1+x^{n}} & x\leq 1\\ 1-\frac{\rho_{\text{c}}}{\alpha_{\text{c}}{\left(x-1\right)}^{2}+x} & x>1 \end{array}$$ (3c)

$$\rho_{\text{c}}=\frac{f_{\text{c}}}{E_{0}\varepsilon_{\text{c}}}$$ (3d)

$$n=\frac{E_{\text{c}}\varepsilon_{\text{c}}}{E_{0}\varepsilon_{\text{c}}-f_{\text{c}}}$$ (3e)

$$x=\frac{\varepsilon }{\varepsilon_{\text{c}}}$$ (3f)

where *E*_0_ is the initial elastic modulus of the concrete; *E*_d_ is the elastic modulus of the damaged concrete; *α*_t_, *α*_c_ are the parameters for the descending branch of the uniaxial tension and compression stress-strain curves, respectively; *f*_c_,*f*_t_ are the uniaxial compression strength and tension strength of the concrete, respectively; *ε*_c_,*ε*_t_ are the strains corresponding with the compression strength *f*_c_ and tension strength *f*_t_, respectively. *d*_c_,*d*_t_ are the parameters for the uniaxial compression and tension damage evolution, respectively. Reference[15] lists the values of all the parameters in Eqs 2A–2E and 3A–3F and the values 33500 MPa, 1.95, 1.36, 0.000107 and 0.00164 were used for *E*_c_, *α*_t_, *α*_c_ *ε*_t_ and *ε*_c_ for C45 level concrete, respectively.

The concrete damaged plasticity model [16] was employed as the constitutive model of the concrete, which was provided in ABAQUS. This model is suitable to simulate the behavior of the concrete under cyclic loading, and can consider the behavior of the damage, crack closure and stiffness recovery of the concrete. Eq 4A–4D [16] refers to this model.

$$\left(1-D\right)=\left(1-s_{\text{t}}D_{\text{c}}\right)\left(1-s_{\text{c}}D_{\text{t}}\right)$$ (4a)

$$s_{\text{t}}=1-w_{\text{t}}r^{*}\;\left(\sigma_{11}\right)\;0\leq w_{\text{t}}\leq 1$$ (4b)

$$s_{\text{c}}=1-w_{\text{c}}\left[1-r^{*}\;\left(\sigma_{11}\right)\right]\;0\leq w_{\text{c}}\leq 1$$ (4c)

$$r^{*}\;\left(\sigma_{11}\right)=H\left(\sigma_{11}\right)=\{\begin{array}{cc} 1 & \sigma_{11}>0\\ 0 & \sigma_{11}<0 \end{array}$$ (4d)

where *D* is the overall damage variable, and the value of *D* is between 0 and 1, which reflects the damage degree of the concrete; *D*_c_ and *D*_t_ are the damage variables under uniaxial compression and tension, respectively; *s*_c_ and *s*_t_ are parameters that are introduced to model stiffness recovery effects associated with stress reversals, especially when the stress state of the concrete changes from tension to compression, causing the tension crack to close. *w*_t_ and *w*_c_ are the weight factors, which are assumed to be material property parameters and remain unchanged during the loading process. If *w*_c_ = 0 or *w*_t_ = 0, there is no stiffness recovery; If *w*_c_ = 1 or *w*_t_ = 1, it means that the stiffness of the concrete fully recovers. However, these two parameters are difficult to measure experimentally. They are generally specified by trial analysis. It was found that the results of the analysis matched the test results well when 0 and 0.3 were used for *w*_t_ and *w*_c_, after many attempts, respectively.

The damage variables *D*_c_ and *D*_t_ under uniaxial compression and tension should be calculated firstly in order to gain the overall damage variable *D*, as shown in Eq 4A. The calculation of *D*_c_ and *D*_t_ of the concrete is based on the principle of energy equivalence[17] in this paper, and assumes that the elastic complementary energy for a damaged material is same in form as that of an undamaged material, except that the stress is replaced by the effective stress in the energy formulation, as shown in Eq 5.

$$W_{\text{d}}^{\text{e}}=W_{0}^{\text{e}}$$ (5)

$$W_{0}^{\text{e}}=\frac{{\tilde{\sigma }}^{2}}{2E_{0}}$$ (6)

$$W_{\text{d}}^{\text{e}}=\frac{\sigma^{2}}{2E_{\text{d}}}$$ (7)

where $W_{0}^{\text{e}}$ and $W_{\text{d}}^{\text{e}}$ are the elastic complementary energies for the undamaged and damaged material, respectively; *σ* and $\tilde{\sigma }$ are the Cauchy nominal stress and effective stress for the concrete, respectively; *E*_0_ and *E*_d_ are the elastic modulus for a undamaged material and the damage elastic modulus for the concrete.

Based on the definition of the damage variable in damage mechanics [18], the relationship between the effective stress and the Cauchy nominal stress is given as follows:

$$\sigma =(1-D_{\text{k}})\tilde{\sigma }\;(\text{k}=\text{t},\text{c})$$ (8)

where k = t and k = c represent that this equation is used for the damage variables under uniaxial tension and compression, respectively.

Combining Eqs 5–8, the damage variables under uniaxial tension and compression can be expressed as Eq 9:

$$E_{\text{d}}=E_{0}{\left(1-D_{\text{k}}\right)}^{2}(\text{k}=\text{t},\text{c})$$ (9)

Considering the stress-strain relationship of the concrete and substituting Eq 2B and Eq 3B into Eq 9, the damage variables under tension and compression can be expressed as Eq 10:

$$D_{\text{k}}=1-{\left(1-d_{\text{k}}\right)}^{1/2}\;(\text{k}=\text{t},\text{c})$$ (10)

A classic bilinear model is adapted for the stress-strain relationship of the rebar. The relationship is given as follows [15]:

$$\sigma_{\text{s}}=\{\begin{array}{ccc} E_{\text{s}}\varepsilon_{\text{s}} & \varepsilon_{\text{s}}\leq \varepsilon_{\text{y}} & \\ f_{\text{y}}+k\left(\varepsilon_{\text{s}}-\varepsilon_{\text{y}}\right) & \varepsilon_{\text{y}}<\varepsilon_{\text{s}}\leq \varepsilon_{\text{u}} & \\ 0 & \varepsilon_{\text{s}}>\varepsilon_{\text{u}} & \end{array}$$ (11)

where the *f*_y_ is the yield strength; *ε*_y_ is the strain for corresponding with the yield strength *f*_y_; *ε*_u_ is the ultimate strain for the rebar; *k* is the slope of the curve after yielding, reflecting the post-peak stiffness; *E*_s_ is the elastic modulus of the rebar. Based on material tests, *ε*_s_, *ε*_u_, *E*_s_ and *k* are equal to 0.0024, 0.088, 190000 MPa and 1900 MPa in this paper, respectively.

### Finite element modeling

Specimen PSC-C-4 and specimen PSC-A-1 were simulated in order to calibrate the numerical models in this paper. Since the effect of the sleeves was limited in the tests, in order to simplify the model, sleeves have not been included. To consider the frictional property of the joint surface in specimen PSC-C-4, contact elements were employed. It is assumed that the magnitude of the pressure stress through the interface is not limited, while tangential behavior obeys Coulomb’s friction law. The critical condition for the relative slip along the contact surfaces is shown as [19]:

$$\tau_{\text{crit}}=\mu p$$ (12)

where *τ*_crit_ is the critical frictional shear stress; *μ* is the frictional coefficient(COF), which is specified as 0.8 in this paper, according to the Chinese Specification [6]; *p* is the pressure stress on the contact surface. When the magnitude of the frictional shear stress is higher than the critical frictional shear stress *τ*_crit_, relative slipping occurs along the contact surfaces. But the critical frictional shear stress *τ*_crit_ is not a constant because of the variation of the pressure stress *p* during the loading process. Specimen PSC-A-1 is the cast-in-situ specimen without a joint, thus no contact elements were used and the foundation elements were tied to the column elements.

**Fig 5:**
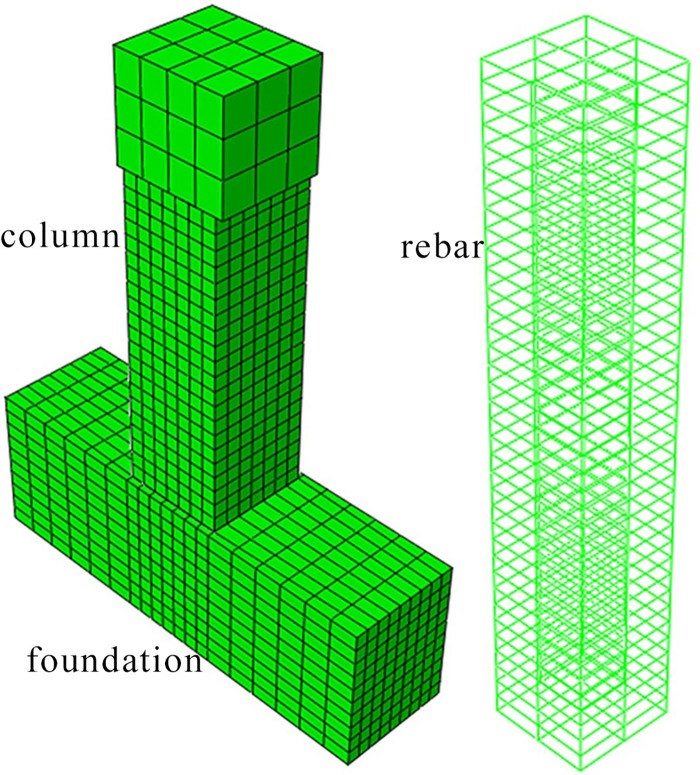
Finite element model.

The rebar was embedded in the concrete when modeling, which meant no relative slip between the concrete and rebar. The element C3D8R [20] was used for the concrete and the element T3D2 [20] was chosen for the rebar. The finite element model is shown in Fig 5.

### Verification of the model

**Fig 6:**
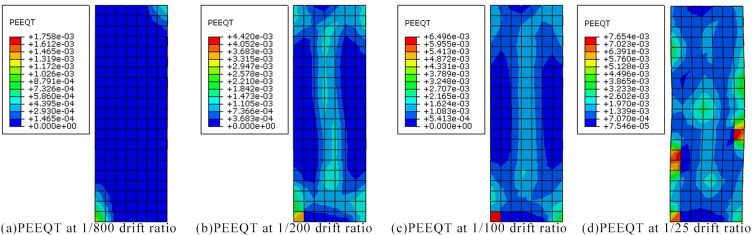
The cloud diagrams of PEEQT for specimen PSC-A-1 under various drift ratios.

Concrete is a brittle material and crack generation and propagation are usually considered when subjected tension stress exceeds the tensile strength. The equivalent plastic strain in uniaxial tension (PEEQT) [16] is generally used to represent the crack development. When the PEEQT is greater than zero, it is assumed that cracks appear in the concrete in the direction perpendicular to the strain. The PEEQT cloud diagrams for specimen PSC-A-1 under various drift ratios are shown in Fig 6. The four figures show the variation of the PEEQT in the analysis during the loading. The blue area (PEEQT = 0) represents that there is no crack. It is observed that cracks occurred at the top and the bottom initially when the drift ratio was around 1/800. With the increase of drift ratio, non-zero PEEQT gradually occurred in the four corners and the mid-column. It implies that crossing cracks formed when the drift ratio reached 1/200. When the drift ratio reached 1/25, the plastic strain had spread to the whole column, leading to an obvious crossing crack pattern eventually occurring.

The development process of the PEEQT for specimen PSC-C-4 is similar to specimen PSC-A-1, and the failure mode of specimen PSC-C-4 is also in diagonal shear. Thus, it is not described repeatedly in this paper. It is found that the failure process and failure modes of the finite element models are similar to the tests.

**Fig 7:**
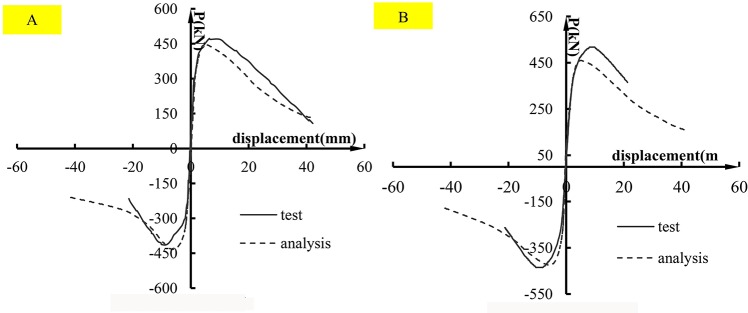
Comparison of skeleton curves of the FE models with that of specimens. (A) specimen PSC-A-1;(B) specimen PSC-C-4.

A comparison of the skeleton curves between the tests and analysis is shown in Fig 7. It is observed that the analyzed skeleton curve of specimen PSC-A-1matches well with the test results. The test curve of specimen PSC-C-4 is a little larger than the analysis curve. The reason could be that the effect of the sleeves was not taken into account.

The shear capacities and the relative errors from the finite element analysis are listed in Table 2. It is observed that the maximum error is less than 15% and indicates satisfactory agreement. Comparing the analysis results with experimental observations, the FE model can predict the failure process of the specimens well. The skeleton curves and the shear capacities of the analysis are similar to the results obtained from the tests. Therefore, the FE model can be further extended to study the joint performance by varying different parameters.

**Table 2:** Shear Capacities of the Test and the Analysis.

Specimens		Capacity of the positive direction	Capacity of the negative direction	Average capacity
**PSC-A-1**	Results of test(kN)	472.70	-406.90	439.80
	Results of analysis(kN)	446.09	-423.29	434.67
	Relative error(%)	5.63	4.03	1.16
**PSC-C-4**	Results of test(kN)	517.70	-438.80	478.25
	Results of analysis(kN)	453.50	-411.06	432.28
	Relative error(%)	12.40	6.32	9.61

## Parametric studies

The axial compression ratio could be an important factor that affects the failure mode of short precast columns. The parameter selected first is the axial compression ratio, which is defined as follows:

$$n=\frac{N}{f_{\text{c}}A}$$ (13)

where *n* is the axial compression ratio of the column, and it is set to 0.6, 0.3, and 0.1 in this paper; *N* is the axial force; *f*_c_ is the compressive strength of the concrete; *A* is the cross section area of the column. Another influencing parameter could be the shear-span ratio, which strongly impacts on the ratio of the normal stress and shear stress. In addition, it may change the failure mode from bending failure to shear failure when the shear-span ratio decreases. In consideration of the boundary condition of the specimens, the shear-span ratio is defined as follows:

$$\lambda =\frac{a}{2h_{0}}$$ (14)

where *λ* is the shear-span ratio, and it is varied as 1.5, 1.0 and 0.5 in this section, respectively; *a* is the height of the column; *h*_0_ is the effective depth of the cross section of the column. By a combination of these two parameters, 9 cases are set out as shown in Table 3, where the title nXX-XX represents n(axial compression ratio)-(shear-span ratio).The material properties assigned are the standard values from reference[15].

**Table 3:** Shear Capacities for the Models.

Cases	Failure mode	Ultimate drift ratio	Analysis results (kN)	Results estimated by Japanese Guideline(kN)	Results estimated by Chinese Specification (kN)
n0.1–1.5	horizontal shear failure	1/428	265.5	343.3	537.54
n0.1–1.0	horizontal shear failure	1/777	218.6	228.5	537.54
n0.1–0.5	horizontal shear failure	1/706	185.1	228.5	537.54
n0.3–1.5	diagonal shear failure	1/64	311.0	520	881.54
n0.3–1.0	diagonal shear failure	1/137	346.2	435.8	881.54
n0.3–0.5	diagonal shear failure	1/287	383.0	327.5	881.54
n0.6–1.5	diagonal shear failure	1/100	363.4	774	1397.54
n0.6–1.0	diagonal shear failure	1/136	376.5	774	1397.54
n0.6–0.5	diagonal shear failure	1/332	490.6	774	1397.54

### Failure modes and criteria

**Fig 8:**
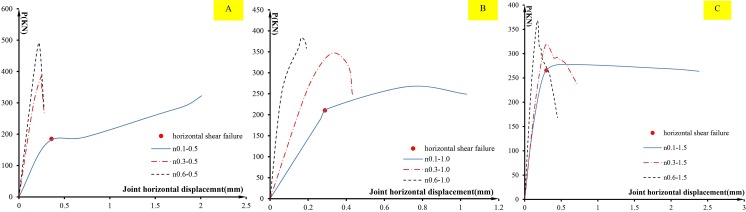
The relationship between the load and the joint horizontal displacement. (A)cases of shear span ratio of 0.5; (B)cases of shear span ratio of 1.0; (c) cases of shear span ratio of 1.5.

**Fig 9:**
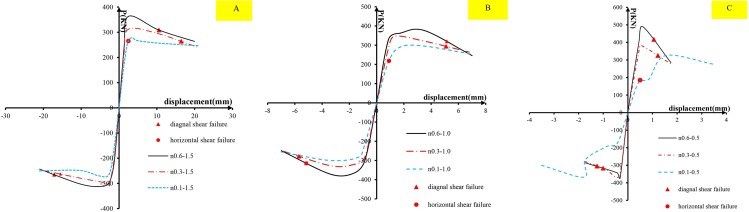
Comparison of skeleton curves with different axial compression ratios. (A)cases of shear span ratio of 1.5; (B) cases of shear span ratio of 1.0; (C) cases of shear span ratio of 0.5.

As discussed in the introduction section, there may be two failure modes for the precast short columns under cyclic lateral load: 1. diagonal shear failure; 2. horizontal shear failure. For the first failure, the damage criteria assumed is that the lateral post-peak load degrades to 85% of the peak load, which means the model can no longer bear the loading and diagonal shear failure occurs. For the second shear failure, if horizontal displacement (slipping) along the joint interface suddenly increases or yields, it is considered that horizontal shear failure occurs. To investigate the failure occurring in the numerical cases, envelope curves of the joint horizontal slipping displacement vs the lateral load are shown in Fig 8, and the skeleton curves of the lateral load and top horizontal displacement are shown in Fig 9. The points corresponding to the two failure modes are marked in Fig 9, and can be used to check the order of the two failure modes. From Fig 8, it is observed that under a low axial compression ratio of 0.1, the joint horizontal displacement increased rapidly for the loads of 185.1 kN, 218.6 kN and 265.5 kN for the cases n0.1–0.5, n0.1–1.0 and n0.1–1.5, respectively, and obvious yielding occurred. The ultimate slipping displacements reached more than 1mm. While under axial compression ratios of 0.3 and 0.6, the joint displacement developed elastically with the horizontal load before diagonal shear damage. The ultimate displacements were less than 0.7 mm. It indicates that no horizontal shear failure occurred on the cases under axial compression ratios of 0.3 and 0.6 before diagonal shear failure. However, the yielding loads for the cases with an axial compression ratio of 0.1 were less than the peak loads on the curves of the top displacement vs loading. It indicates that horizontal shear failure has occurred on these cases prior to diagonal shear failure. In other words, cases with an axial compression ratio of 0.1failed in horizontal shear, and cases with axial compression ratios of 0.3 and 0.6 failed in diagonal shear. It proves that the axial compression ratio is one of the important factors to impact on the failure mode of short precast concrete columns.

### Ductility behavior and shear capacity

To investigate the effects of the axial compression ratio and shear-span ratio on the ductility of precast columns, the ultimate horizontal displacements of all cases are listed in Table 3, where the horizontal displacement is represented in the drift ratio, since the column heights are different. Many studies[21] have shown that the ductility for cast-in-situ columns decreases as the axial compression ratio increase. The analysis shows a similar trend to the precast columns except for the columns with an axial compression ratio of 0.1, as shown in Table 3. However, it is observed that the ultimate drift ratios for the columns under an axial compression ratio of 0.1, of which the failure mode is horizontal shear failure, are very limited and much lower than the columns under axial compression ratios of 0.3 and 0.6. It indicates that horizontal shear failure shows very brittle behavior.

**Fig 10:**
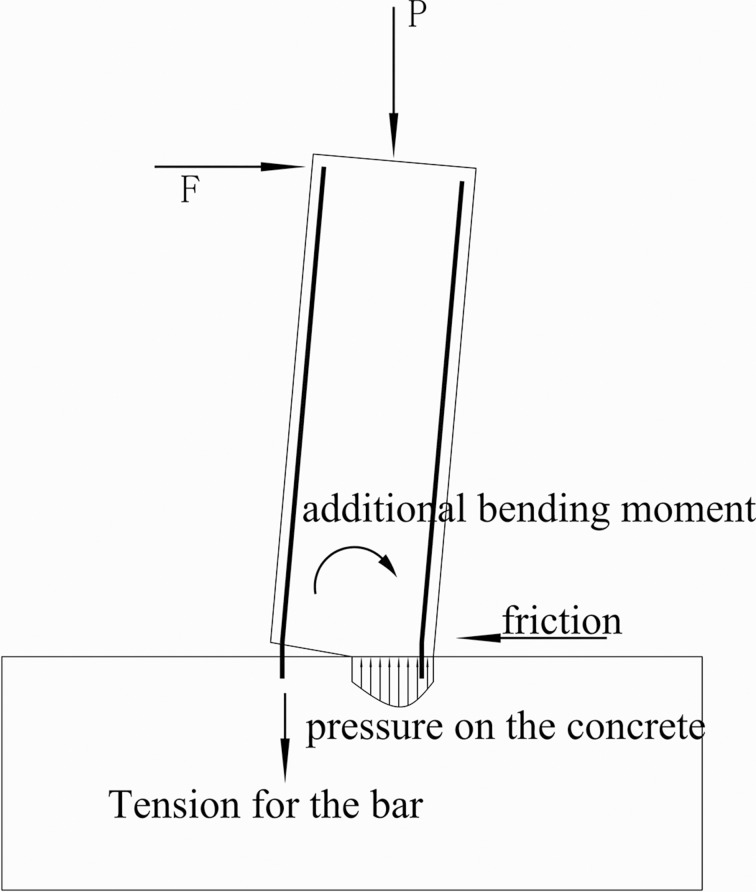
Force transfer along the joint.

Table 3 lists the shear capacities of the cases obtained from the analysis. When diagonal shear failure occurs, the ultimate shear strength is specified as the peak load of the skeleton curve; while the horizontal shear failure occurs, the strength is defined as the yield loading, shown in Fig 8. Although the columns with horizontal shear failure can continually resist external horizontal loads after yielding (shown in Fig 9), the joint displacement or slipping results in the integrity decreased, which is important for precast structures. From Table 3, it can be seen that the ultimate strength decreases with the axial compression ratio decease. Under the same axial compression ratio, the ultimate strength increases with the shear-span ratio decrease for the cases with the medium and high axial compression ratios. However, under low axial compression ratio when horizontal shear failure occurs, the ultimate strength decreases with shear-span decrease, which means that the shear-span ratio benefits to the strength of the horizontal joint. Fig 10 shows the internal force distribution on the horizontal joint. The bending moment causes additional compression stress, which determines the magnitude of the friction. When the shear span ratio is low, the bending moment and further friction is small. Therefore, the shear strength decreases with the shear span ratio for horizontal shear failure columns.

## Estimating the shear capacity of horizontal joints

In this section, both the Chinese Specification[6] and the Japanese Guideline[12] were used to estimate the shear capacity of the horizontal joint. In the Chinese Specification[6], the shear capacity equation of the horizontal joint is expressed as Eq 15A and 15B, where Eq 15A is for the column bearing pressure and Eq 15B is for the columns bearing tension force.

$$V_{\text{uE}}=0.8N+1.65A_{\text{sd}}\sqrt{f_{\text{c}}f_{\text{y}}}$$ (15a)

$$V_{\text{uE}}=1.65A_{\text{sd}}\sqrt{f_{\text{c}}f_{\text{y}}\left[1-{\left(\frac{N}{A_{\text{sd}}f_{\text{y}}}\right)}^{2}\right]}$$ (15b)

where *V*_uE_ is the shear capacity of the horizontal joint of the precast columns; *N* is the axial force; *A*_sd_ is the sectional area of the rebar passing through the joint.

However, in the Japanese Guideline[6], the shear capacity of the horizontal joint is more conservative, and is expressed as Eq 16A–16E.

$$V_{\text{c}}=\mu C$$ (16a)

$$V_{\text{do}}=1.65A_{\text{se}}\sqrt{f_{\text{c}}f_{\text{y}}\left(1-\alpha^{2}\right)}$$ (16b)

$$\alpha =\sigma_{\text{s}}/f_{\text{y}}\;\alpha \leq 1.0$$ (16c)

$$V_{\text{uE}}=\max\left(V_{\text{c}}\;,\;V_{\text{do}}\right)$$ (16d)

$$\begin{array}{l} C=\frac{M}{j_{\text{t}}}+\frac{N}{2}\;\mathrm{for}\frac{\left|M\right|}{2}\geq \frac{\left|N\right|}{2}\\ C=N\;\mathrm{for}\frac{\left|M\right|}{2}\leq \frac{\left|N\right|}{2} \end{array}$$ (16e)

Where *μ* is the COF of the surface; *V*_c_ is the shear force transmitted by the friction; *V*_do_ is the shear force transmitted by the dowel action of the rebar; *M* is the bending moment along the joint; *N* is the axial load; *α* is the ratio of stress to yield strength for the rebar; *A*_se_ is the effective areas of the rebar, which is usually the area of the rebar in the compressional zone.

The key difference between both specifications is whether to sum the effects of the dowel action and friction. In the Japanese Guideline[12], the shear capacity takes the maximum between the dowel action and friction, while the Chinese Specification[6] takes the summation. The Japanese Guideline[12] considers that the deformation is different when the two load paths reach the ultimate capacity, so the shear capacity takes the larger value of the two. There is also an additional difference in estimating the dowel action of the rebar between the two specifications. All the rebar is taken into account in the Chinese Specification, but the Japanese Guideline usually takes into account the rebar in the compression zone only.

It is observed that the bending moment is taken into account in Eq 16A–16E, and the bending moment is usually determined by the shear-span ratio and the lateral force, as shown in Fig 10. Thus the Japanese Guideline[6] has taken into account indirectly the influence of the shear-span ratio. However, the shear-span ratio is not considered in Chinese Specification. As discussed in preceding part of the paper, the shear-span ratio indeed was an effect on the shear capacity of the horizontal joint, thus the Japanese Specification is more reasonable.

The shear capacities of the horizontal joint estimated by the Chinese Specification[6] and the Japanese Guideline[12] are compared with the analysis ultimate capacities in Table 3. It is noted that only the cases with an axial compression ratio of 0.1 failed along the horizontal joint. Thus the capacities of the three cases represent the shear capacities of the horizontal joints, while other cases represent the diagonal shear capacities. It is observed that the capacities estimated by the Chinese Specification are much higher than the analysis results and the maximum error is190.4%.The results estimated by Japanese Guideline also overestimate the shear capacities of the horizontal joints, but are much closer to the analysis results with the maximum error 29.3%.

## Conclusions

In this paper, four specimens were tested to study the shear behavior of precast columns with sleeve grouted connections under cyclic loading. Based on the test specimens, finite element models have been established and extended for parametric study using the software ABAQUS. Through the numerical analysis, the effect of the axial compression ratio and shear-span ratio were investigated. Based on the results of the testing and finite element analysis, the following conclusions can be drawn:

1. The sleeve grouted connection does not obviously reduce the capacity and the ductility of the precast short column, and both the joint configuration with shear keys and natural joint interfaces can ensure shear force transmission. Thus, the sleeve grouted connection shows good behavior, equivalent to the cast-in-situ connection under the same test condition.
2. FE analysis indicates that the axial compression ratio is an important factor for the failure mode of precast concrete columns. Under medium and high axial compression ratios, diagonal shear failure will occur in the precast short columns. While under low axial compression ratios, i.e. 0.1, horizontal shear failure, which is shown as a large or sudden slipping along the joint, will occur.
3. FE analysis indicates the ductility of the precast column with horizontal shear failure is very limited and lower. It indicates brittle performance for the precast column if horizontal shear failure occurs. Therefore such failure should be avoided.
4. FE analysis indicates that the joint shear capacity increases with the shear-span ratio. It is because the bending moment improves the pressure through the interfaces and increases the friction. Therefore the effect of the shear-span ratio on joint shear capacity should be taken into account when estimating the capacity.
5. By comparing the Chinese Specification and Japanese Guideline for precast structures, the shear capacity of the horizontal joint estimated by the Japanese Guideline is much closer to those in our analysis than the Chinese Specification. However, both overestimate the shear capacity of the horizontal joint.

## Supporting Information

S1 File Data of the skeleton curve for the four specimens. (XLSX)

S2 File Data of the skeleton curve for the analysis cases. (XLSX)

S3 File The joint horizontal displacement data. (XLSX)
